# Zhachong Shisanwei pill drug-containing serum protects H_2_O_2_-Induced PC12 cells injury by suppressing apoptosis, oxidative stress via regulating the MAPK signaling pathway

**DOI:** 10.3389/fphar.2024.1445597

**Published:** 2024-10-09

**Authors:** Hanqiong Hu, Yifan Sun, Zhen Yang, Limuge Che, Mingyang Cai, Xiaoxuan Li, Xianju Huang, Hurile Bagen, Wulan Qiqige, Wuyunsiri Guleng, Liqun Ma, Haiying Tong

**Affiliations:** ^1^ College of Traditional Chinese Medicine, Beijing University of Chinese Medicine, Beijing, China; ^2^ Institute of Ethnic Medicine, Beijing University of Chinese Medicine, Beijing, China; ^3^ Medicine Innovation Center for Nationalities, Inner Mongolia Medical University, Hohhot, China; ^4^ Hospital of Pediatrics, The First Affiliated Hospital of Henan University of Traditional Chinese Medicine, Zhengzhou, China; ^5^ Pediatric Medical College, Henan University of Traditional Chinese Medicine, Zhengzhou, China; ^6^ College of Pharmaceutical Science, South-Central Minzu University, Wuhan, China; ^7^ Mongolian Medical College, Inner Mongolia Medical University, Hohhot, China; ^8^ Psychosomatic Medicine Department, Inner Mongolia International Mongolian Hospital, Hohhot, China

**Keywords:** Zhachong shisanwei pill (ZSP), ischemic stroke (IS), oxidative stress, PC12 cells, MAPK pathway

## Abstract

**Introduction:**

Zhachong Shisanwei Pill (ZSP) is a classical Mongolian formula that combines 13 types of Chinese medicinal materials and has been used for treating ischemic stroke (IS) for centuries. However, the underlying molecular mechanisms have yet to be fully elucidated. The aim of this study is to explore potential mechanism of ZSP on nerve cells in cerebral ischemic injury.

**Methods:**

To simulate the pathological process of oxidative stress following IS, an injury model using PC12 cells was induced with hydrogen peroxide (H_2_O_2_). Afterward, PC12 cells were treated with ZSP medicated serum at low, medium, and high doses. Various assays were conducted to assess cell viability and oxidative stress indicators, including lactate dehydrogenase (LDH), malondialdehyde (MDA), superoxide dismutase (SOD), catalase (CAT), reactive oxygen species (ROS), and mitochondrial membrane potential (MMP). Cell apoptosis was evaluated through morphological assessment and flow cytometry. Additionally, the expression levels of apoptosis-related proteins (Bcl-2, Bax, Caspase-9, Caspase-3, PARP) and signaling pathway proteins (JNK, phosphorylated JNK, ERK, phosphorylated ERK, p38, and phosphorylated p38) were measured using automated Western blotting.

**Results:**

Our findings indicate that ZSP medicated serum preconditioning improves the condition of PC12 cells injured by H_2_O_2_. Specifically, it increased cell survival rates and reduced LDH release. Additionally, ZSP treatment decreased ROS levels and MDA content, while enhancing the activity of SOD and CAT in the injured PC12 cells. ZSP also reversed the depolarization of mitochondrial membrane potential and protected cells from apoptosis by modulating the expression of apoptosis-related proteins, including Bcl-2, Bax, Caspase-9, Caspase-3, and PARP. Furthermore, the overactivation of the MAPK signaling pathway due to H_2_O_2_-induced injury was inhibited, as evidenced by the downregulation of phosphorylated JNK, ERK, and p38 levels.

**Discussion:**

Mongolian medicine ZSP demonstrates protective effects against H_2_O_2_-induced oxidative stress and apoptosis in PC12 cells. The underlying mechanism may involve the inhibition of the MAPK signaling pathway, enhancement of antioxidant enzyme activity, reduction of intracellular peroxidation levels, and suppression of intrinsic apoptosis pathways.

## 1 Introduction

Stroke is defined as a cerebrovascular disease caused by the rupture or blockage of cerebral blood vessels, characterized by sudden onset and rapid development of localized or diffuse brain deficits. Ischemic stroke (IS) is initiated by occlusion of cerebral arteries, leading to local hypoxia and subsequent damage to brain tissues (Barthels and Das, 2020). IS is associated with high morbidity, mortality, recurrence rates, and costs. In 2019, of the 3.94 million stroke cases in China, 2.87 million were ischemic strokes (Ma et al., 2021), and the global economic burden of IS was estimated at $964.51 billion (Gerstl et al., 2023). Advances in pharmacological and mechanical thrombolytic recanalization therapies have facilitated some progress in aiding patient recovery from ischemic stroke (Zheng et al., 2023; Sedghi et al., 2024). However, in clinical practice, despite successful revascularization of occluded arteries, nearly half of the patients fail to achieve favorable clinical outcomes (Nie et al., 2023; Sperring et al., 2023). It is noteworthy that a significant proportion of stroke survivors experience post-stroke sequelae, including paralysis, depression, aphasia, and visual impairment (Das and Rajanikant, 2018; Vitti and Hillis, 2021; Smith MC et al., 2022).Therefore, it is imperative to enhance the recovery of neurological function in patients with ischemic stroke and other associated sequelae.

Following IS onset, excitotoxicity, oxidative stress, and neuroinflammatory episodes are immediately elicited within the brain, with the latter being central to secondary brain injury (Cui et al., 2021). Cerebral ischemia-reperfusion (I/R) injury is accompanied by complex signaling processes, including blood-brain barrier disruption, inflammation, mitochondrial dysfunction, oxidative stress, and apoptosis (Jaganjac et al., 2022), which can exacerbate neuronal damage and lead to severe functional defects. The ischemic brain is highly susceptible to oxidative damage owing to its high consumption of oxygen, rich content of iron and unsaturated lipids, and relatively low endogenous antioxidant capacity (Chamorro et al., 2016). The disequilibrium between the production of prooxidants and the scavenging ability of the antioxidant defense system contributes to a state of oxidative stress (Sies, 2015). Ischemia-induced vascular obstruction and I/R after thrombolytic therapy both produce excessive reactive oxygen species (ROS) (Alsbrook et al., 2023). The aforementioned injuries further cause severe damage to brain tissues (Huang et al., 2015). Meanwhile, the subsequent complex molecular cascade events will further aggravate the process of oxidative stress. It is well-known that the overproduction of ROS and the accompanying oxidative stress play an essential part in the pathogenesis of ischemic injury (Qin et al., 2022; Huang et al., 2022; Chen et al., 2020). Under physiological conditions, ROS can act as signaling molecules and are widely involved in maintaining homeostasis, metabolism, growth, and differentiation *in vivo* (Zhu et al., 2022; Lyle and Griendling, 2006; Dröge, 2002). However, high concentrations of ROS can cause tissue destruction and cell damage. Excessive ROS could further attack the brain, leading to lipid and protein peroxidation, and eventually cause immeasurable damage (Chen et al., 2011). Oxidative stress-induced neuronal apoptosis plays a pivotal role in the pathogenesis of ischemic brain injury. In apoptosis, ROS play a critical role by initiating various signal transduction pathways, including the activation of both intrinsic and extrinsic caspase family proteins. This activation can result in the overexpression of apoptotic and inflammatory genes, contributing to excessive cellular damage and inflammation. Therefore, therapeutic strategies aimed at regulating apoptotic processes and oxidative stress are considered crucial for mitigating IS damage and facilitating recovery.

Zhachong Shisanwei Pill (ZSP), a classical Mongolian medicinal formula, consisting of thirteen traditional medicinal herbs, has the effects of relaxing the tendons and activating the blood, calming and tranquilizing the mind. Consequently, it is widely used by the Mongolian people in China as an adjuvant treatment for IS. YING conducted a clinical study evaluating the efficacy of ZSP in combination with vinpocetine for treating stroke sequelae, the results indicated that the observation group experienced significantly greater improvements in motor function scores compared to the control group, additionally, scores on the Self-Rating Anxiety Scale, the Hamilton Depression Rating Scale, and the Mini-Mental State Examination demonstrated a similar trend of improvement (YING and Xueqin, 2020). Moreover, experiments conducted in rats with middle cerebral artery occlusion model demonstrated that ZSP modulates the lysosomal pathway, providing protection against IS by downregulating LAMP2 and AP3M1, and upregulating SCARB2 (Song Q et al., 2023). Previously, our group conducted a review and clinical positioning study on this formula to clarify its therapeutic advantages for IS. However, there is limited research on the effect of ZSP on nerve cells, especially on the molecular level.

In this study, we aimed to investigate the protective effects of ZSP in nerve cells and elucidate its potential mechanisms by assessing apoptosis, oxidative stress indicators, mitochondrial function, and relevant cellular signaling pathways. This research provides a theoretical foundation for the therapeutic application of ZSP in ischemic IS and contributes to the knowledge base for the development and utilization of Mongolian medicinal resources.

## 2 Materials and methods

### 2.1 ZSP

ZSP is derived from the Pharmaceutical Standards of the Ministry of Health of the People’s Republic of China: Mongolian Medicine *Volume* (Pharmacopoeia Commission of PRC, 1998). ZSP is formulated by combining 13 types of Chinese medicinal materials with a water-based pill. We verified the plant names with https://www.worldfloraonline.org on 19 September 2023 and listed them in Table 1. ZSP (national drug approval number: Z15020409, lot number: 1908003) was procured from Inner Mongolia Mongolian Medicine Co., Ltd. (Nei Mongol, China). The pills are pulverized into fine powder, sieved using a No. 7 sieve, and subsequently dissolved in water to formulate solutions for different dosage levels to gavage.

**TABLE 1:** The sources and constituents of ZSP.

Crude drugs	Vernacular name			Source
	Han name	Mongolian name	English name	
Fructus Terminaliae Bellirica	诃子	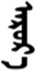	Myrobalan fruit	Terminalia chebula Retz.
	He-zi	Arur		
Radix Aconiti kusnezoffii	制草乌	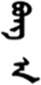	Prepared Kusnezoff Monkshood Root	Aconitum kusnezoffii Rchb.
	Zhi-Cao-wu	Bong aa		
Acori Tatarinowii Rhizoma	石菖蒲	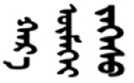	Grassleaf Sweetflag Rhizome	Acorus tatarinowii Schott.
	Shi-chang-pu	Har umhei zegs		
Radix Aucklandiae	木香	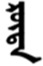	Common Aucklandia Root	Aucklandia lappa Decne
	Mu-xiang	Ruuda		
Moschus	麝香	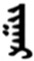	Musk	Artificial musk (Beijing Lianxin pharmaceutical Co., Ltd., 2021YR148)
	She-xiang	Zaar		
Corallium	珊瑚	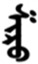	Coral	*Corallium japonicum* Kishinouye
	Shan-hu	Sur		
Margarita	珍珠	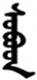	Margarite	*Pteria martensii* (Dunker), *Hyriopsis cumingii* (Lea), *Cristaria p1icata* (Leach)
	Zhen-zhu	Subd		
Semen Myristicae	肉豆蔻	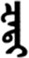	Nutmeg	Myristica fragrans Houtt.
	Rou-dou-kou	Zadi		
Flos Caryophylli	丁香	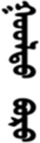	Clove	Caryophyllus aromaticus L.(syn. Eugenia caryophyllata Thunb.)
	Ding-xiang	Goolt bor		
Lignum Aquilariae Resinatum	沉香	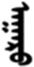	Chinese Eaglewood	Agallochum grandiflorum (Benth.) Kuntze
	Chen-xiang	Agruu		
Limonitum	禹粮土	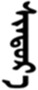	Limonitum	Limonite
	Yu-liang-tu	Sendraa		
Magnetitum	磁石	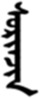	Magnetitum	Lodestone
	Ci-shi	Soronzon		
Glycyrrhizae Radix et Rhizoma	甘草	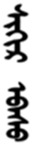	Liquorice	Glycyrrhiza uralensis Fisch. Glycyrrhiza inflata Batalin. Glycyrrhiza glabra L.
	Gan-cao	Siherobs		

### 2.2 Animals and cells

Twenty 8–10 weeks old male SPF SD rats, 250 ± 20 g, were provided by Spelford (Beijing) Biotechnology Co., Ltd. [License No. SYXK (Beijing) 2020-0033]. Animals were treated in accordance with the US NIH guidelines and the requirements of the Animal Ethics Committee of Beijing University of Chinese Medicine (license number: BUCM-4-2022081601-3053). The animals were housed in a standard laboratory animal facility under controlled environmental conditions: temperature maintained between 20°C–25°C, relative humidity at 40%–60%, and a 12-h light/dark cycle. Food and water were provided *ad libitum*.

Rat pheochromocytoma cells (PC12 cells, highly differentiated) were obtained from the American Type Culture Collection (ATCC) and purchased from Beijing Beina Chuanglian Biotech Institute.

### 2.3 Materials and reagents

H_2_O_2_ (H433851) was obtained from Aladdin Biochemical Technology Co., Ltd. (Shanghai, China). DMEM (C11995500BT) and TRYPSIN 0.25% EDTA (25200056) were purchased from Invitrogen (Carlsbad, CA, United States). Penicillin-Streptomycin Solution (C100C8) was sourced from New Cell & Molecular Biotech Co., Ltd. (Suzhou, Jiangsu, China). Fetal Bovine Serum (FBS, AQMV09900), Cell Counting Kit-8 (CCK-8, AQ308-500T), Radioimmunoprecipitation assay buffer (RIPA, AQ522), and Bicinchoninic Acid Protein Assay Kit (BCA, AQ526-500T) were acquired from Beijing Aoqing Biotechnology Co., Ltd. (Beijing, China). Malondialdehyde (MDA, S031S), superoxide dismutase (SOD, A00-3-2), and lactate dehydrogenase (LDH, A020-1-2) assay kits were obtained from Nanjing Jiancheng Bioengineering Institute (Nanjing, Jiangsu, China). Reactive oxygen species (ROS, S0033S) assay kit and Hoechst33258 assay kit (C011) were purchased from Beyotime Biotechnology Co., Ltd. (Shanghai, China). The FITC Annexin V Apoptosis Detection Kit (556547) was sourced from BD Pharmingen (San Diego, CA, United States). The Mitochondrial Membrane Potential (MMP) assay kit with JC-1 (M8650) was obtained from Beijing Solarbio Science & Technology Co., Ltd. (Beijing, China). Primary antibodies against Bcl-2 (ab196495), Bax (ab32503), caspase-9 (ab184786), ERK1/2 (ab184699), and JNK (ab179461) were purchased from Abcam (Cambridge, UK). Primary antibodies against caspase-3 (14220T), PARP (9532T), phospho-ERK1/2 (4370T), phospho-JNK (4668T), p38 (8690), and phospho-p38 (4511T) were obtained from Cell Signaling Technology (Beverly, MA, United States). The primary antibody against GAPDH was acquired from Proteintech (Chicago, IL, United States).

### 2.4 Preparation of drug-containing serum of ZSP

After 1 week of adaptive feeding, rats were randomly assigned to four groups: a control group (receiving only purified water), a low-dose ZSP group (90 mg/kg/d), a middle-dose ZSP group (180 mg/kg/d), and a high-dose ZSP group (360 mg/kg/d). The dosage for the middle-dose ZSP group was determined using the “direct conversion method of equivalent dose,” based on clinical dosing guidelines. In the low-dose and high-dose groups, the ZSP dosage was set at half and twice the equivalent dose, respectively. All groups were administered ZSP orally via gavage for a period of 7 days. One hour after the final gavage, the rats were anesthetized using pentobarbital, and blood samples were collected from the abdominal aorta. The blood samples were centrifuged at 3,000 rpm for 20 min, after which the upper layer of clear yellowish serum was collected. The serum was then heated at 56°C for 30 min to inactivate, filtered and sterilized by 0.22 μm microporous membrane and stored at −80°C.

### 2.5 Cell culture and treatment

PC12 cells were cultured in culture flasks or dishes using DMEM medium supplemented with 10% FBS and 1% antibiotic solution (100 U/mL penicillin and 100 μg/mL streptomycin) at 37°C in an incubator with a humidified atmosphere of 95% O₂ and 5% CO₂. The culture medium was refreshed daily based on the growth status of the cells. When confluence approached 80%–90%, subculturing was conducted. Typically, subculturing was performed every other day at a 1:3 split ratio. To determine the appropriate oxidative stress conditions, PC12 cells were exposed to 100–800 μM H₂O₂ for 1 h. After identifying the suitable concentrations for medicated serum administration, the cells were divided into five groups: a control group (cells were cultured with normal rat serum), a model group (cells were cultured with H₂O₂ and normal rat serum), a low-dose ZSP group (cells were cultured with H₂O₂ and low-dose ZSP containing serum, abbreviated as L-ZSP), a medium-dose ZSP group (cells were cultured with H₂O₂ and medium-dose ZSP containing serum, abbreviated as M-ZSP), and a high-dose ZSP group (cells were cultured with H₂O₂ and high-dose ZSP containing serum, abbreviated as H-ZSP).

### 2.6 Assessment of cellular status

Cell viability was evaluated using the Cell Counting Kit-8 (CCK-8) assay. Briefly, PC12 cells were seeded at a density of 1 × 10⁴ cells per well and cultured for 24 h. Subsequently, the cells were treated with ZSP-containing serum for 12 h, followed by exposure to H₂O₂ for 1 h. To assess viability, 10 μL of CCK-8 reagent was added to each well, and the plates were incubated for 1.5 h at 37°C. Absorbance was measured at 450 nm using a microplate reader (BIO-RAD, United States). Cell viability was calculated as a percentage relative to the control group.

Cellular damage was assessed using LDH (lactate dehydrogenase) assay. After being exposed to drug-containing serum of ZSP and H_2_O_2_, the cell supernatant was collected. After sequentially adding the reagents according to the manufacturer’s instructions, the samples were incubated at room temperature for 5 min. The optical density (OD) was then measured at a wavelength of 450 nm using a microplate reader.

### 2.7 Measurement of oxidative stress

This experiment aimed to evaluate oxidative stress by measuring levels of SOD, MDA, CAT, ROS. PC12 cells were seeded at a density of 1.2 × 10⁶ cells per well and incubated for 24 h. Following treatment with ZSP-containing serum and H₂O₂ as previously described, the cells were harvested and sonicated in PBS to produce cell homogenates. Protein concentrations in the lysis buffer were quantified using the BCA protein assay kit. MDA, CAT, SOD activities were measured according to the manufacturer’s protocols. After various pretreatments, as described earlier, the cells were collected by centrifugation and incubated with diluted DCFH-DA (a kind of ROS fluorescent probe) for 20 min in the dark at 37°C. The cells were then washed three times with PBS. Intracellular ROS levels were analyzed by measuring the fluorescence intensity of DCF using a flow cytometer (BD Company, United States).

### 2.8 Cell apoptosis evaluation

In this study, cell apoptosis was assessed using multiple techniques. The JC-1 assay was employed to detect MMP changes, while the Hoechst 33258 fluorescence staining method was utilized to observe morphological changes indicative of apoptosis. Additionally, the Annexin V-FITC/PI dual staining method was used to quantify the apoptosis rate. These methods collectively provided a comprehensive evaluation of apoptosis in the cell populations studied.PC12 cells from various experimental groups were washed with DMEM and incubated with JC-1 dye (1 μM) in DMEM at 37°C for 20 min. After incubation, the cells were washed with incubation buffer and analyzed using an LSRFortessa™ flow cytometer (BD Company, United States). Red and green fluorescence emissions were detected using the FL2 and FL1 channels, respectively. The ratio of these fluorescence signals was utilized to assess the extent of mitochondrial membrane potential depolarization. For morphological changes, PC12 cells from different treatment groups were washed, fixed with paraformaldehyde, and stained with Hoechst 33258 for 5 min in the dark at 37°C, following the manufacturer’s protocol. Nuclear changes indicative of apoptosis were observed using an Olympus fluorescence microscope (Tokyo, Japan) at an excitation wavelength of 461 nm. For apoptosis quantification, PC12 cells were collected and centrifuged to remove the supernatant. The cell pellets were resuspended in 500 μL of binding buffer and stained with 5 μL of Annexin V-FITC and 5 μL of propidium iodide for 15 min at room temperature. Apoptotic cells were then quantified using a FACSCanto II flow cytometer (BD Corporation, United States).

### 2.9 Automated western blot and analysis

PC12 cells were lysed using RIPA buffer containing protease and phosphatase inhibitors on ice for 30 min. The supernatant was collected by centrifugation at 12,000 rpm for 15 min at 4°C. Protein concentration was determined using a BCA assay kit. Automated Western blotting was performed using the Wes™ system (ProteinSimple, United States). All reagents were prepared and utilized according to the manufacturer’s instructions. Primary antibodies were diluted using Antibody Diluent 2 included in the kit, with the following dilution ratios: 1:100 for Bcl-2, Bax, Caspase-9, Caspase-3, PARP, JNK, p-JNK, p38, and p-p38; 1:500 for Cyt-c; 1:1000 for Erk1/2; 1:200 for p-Erk1/2; and 1:2000 for GAPDH. The biotinylated ladder, primary antibody, streptavidin-horseradish peroxidase (streptavidin-HRP), secondary HRP conjugate, and luminol-peroxide mix which were provided with the Wes™ ProteinSimple Kit were sequentially loaded onto the Wes separation module (12–230 kDa prefilled plate). The plate and capillary tube were then placed in the system for automated operation for 3 h. The intensities of the acquired chemiluminescence signals were quantified using Compass software (ProteinSimple, United States).

### 2.10 Statistical analysis

Data analysis and standard deviation computation were performed using SPSS version 23.0, while statistical graphs were generated using GraphPad Prism version 9.0.0. Results are expressed as mean ± SEM. One-way ANOVA was employed to compare multiple independent data groups, and *post hoc* comparisons between groups were conducted using the LSD test. A *p*-value of less than 0.05 was considered statistically significant.

## 3 Results

### 3.1 ZSP-containing serum exhibits protective effects on H_2_O_2_-injured PC12 cells

**FIGURE 1:**
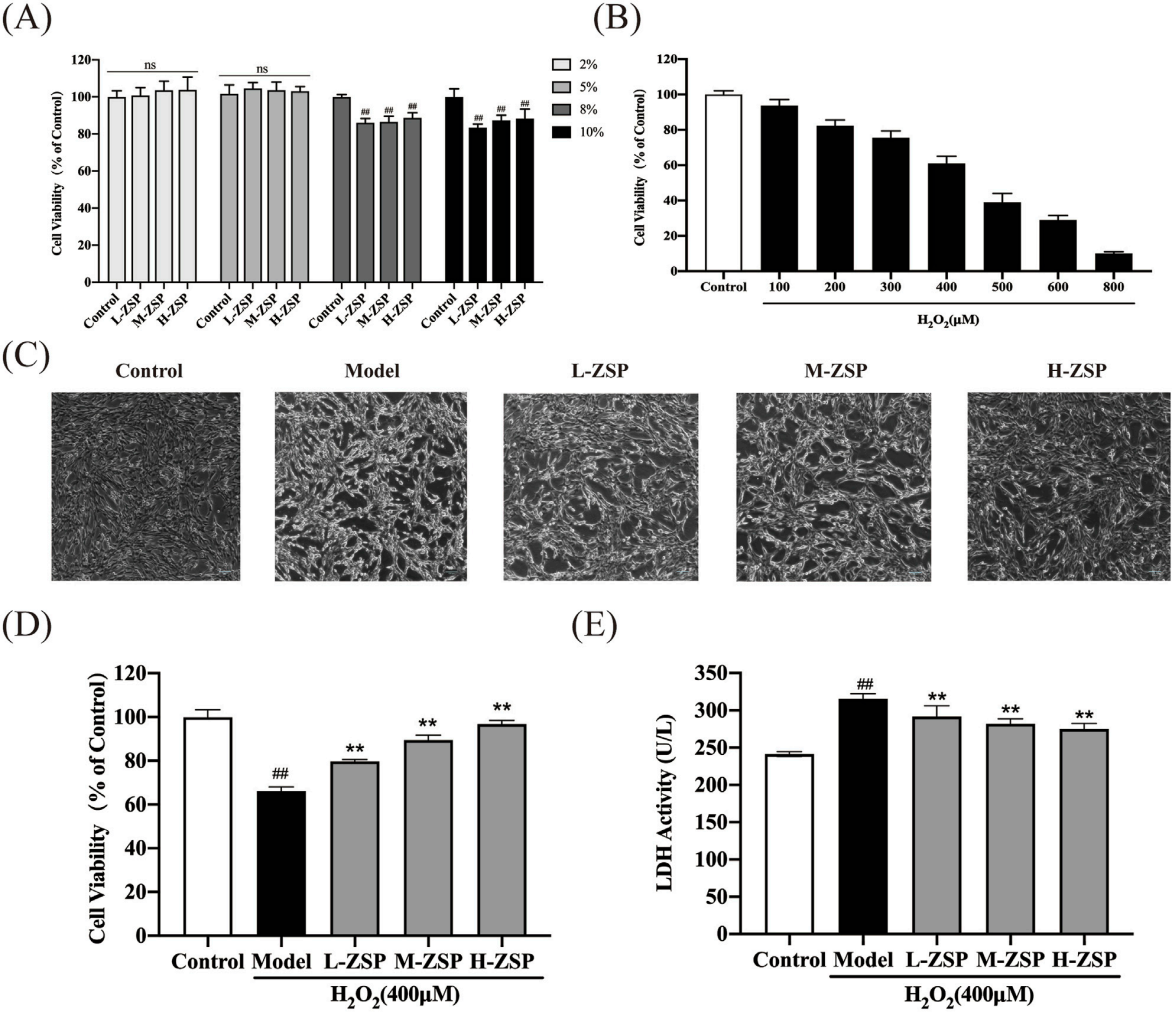
Protective effect of ZSP-containing serum on H_2_O_2_-induced PC12 cells injury. **(A)** Effect of different concentrations of ZSP-containing serum on PC12 cell viability. n = 6 per group. **(B)** The cell viability influenced by different concentrations of H_2_O_2_ for 1 h n = 6 per group. **(C)** Morphology and amount of cells in each group (×100). **(D)** Effect of ZSP-containing serum on the cell viability of H_2_O_2_-induced PC12 cells injury. n = 6 per group. **(E)** Effect of ZSP-containing serum on LDH activity of H_2_O_2_- treated PC12 cells. n = 3 per group. ^#^ *p* < 0.05 and ^##^ *p* < 0.01, *versus* the control group. ^*^ *p* < 0.05 and ^**^ *p* < 0.01, *versus* the model group.

To determine the optimal concentration of ZSP-containing serum for administration to PC12 cells, we conducted CCK-8 assays. Toxic and inhibitory effects on cell growth were observed at serum concentrations of 8% and 10% (*p* < 0.01). Consequently, a 5% serum concentration was selected for subsequent experiments to ensure the safety of the ZSP-containing serum (Figure 1A). Cell viability was reduced in a dose-dependent manner in PC12 cells following incubation with H_2_O_2_ (100–800 μM) for 1 h (Figure 1B). Exposure to 400 μM H_2_O_2_ decreased the survival rate of PC12 cells to 60%. There was a marked decline in cell morphology and number in the H_2_O_2_-treated group, however, ZSP-containing serum treatment improved both the morphology and quantity of cells (Figure 1C). Additionally, cell viability decreased and LDH levels increased in the H_2_O_2_ model group, whereas treatment with ZSP-containing serum significantly rescued cell viability and LDH production significantly (Figures 1D,E). These results suggest that ZSP-containing serum has a protective effect on PC12 cells against H_2_O_2_-induced injury.

### 3.2 ZSP-containing serum significantly reduced H_2_O_2_-induced oxidative stress and intercellular ROS release

**FIGURE 2:**
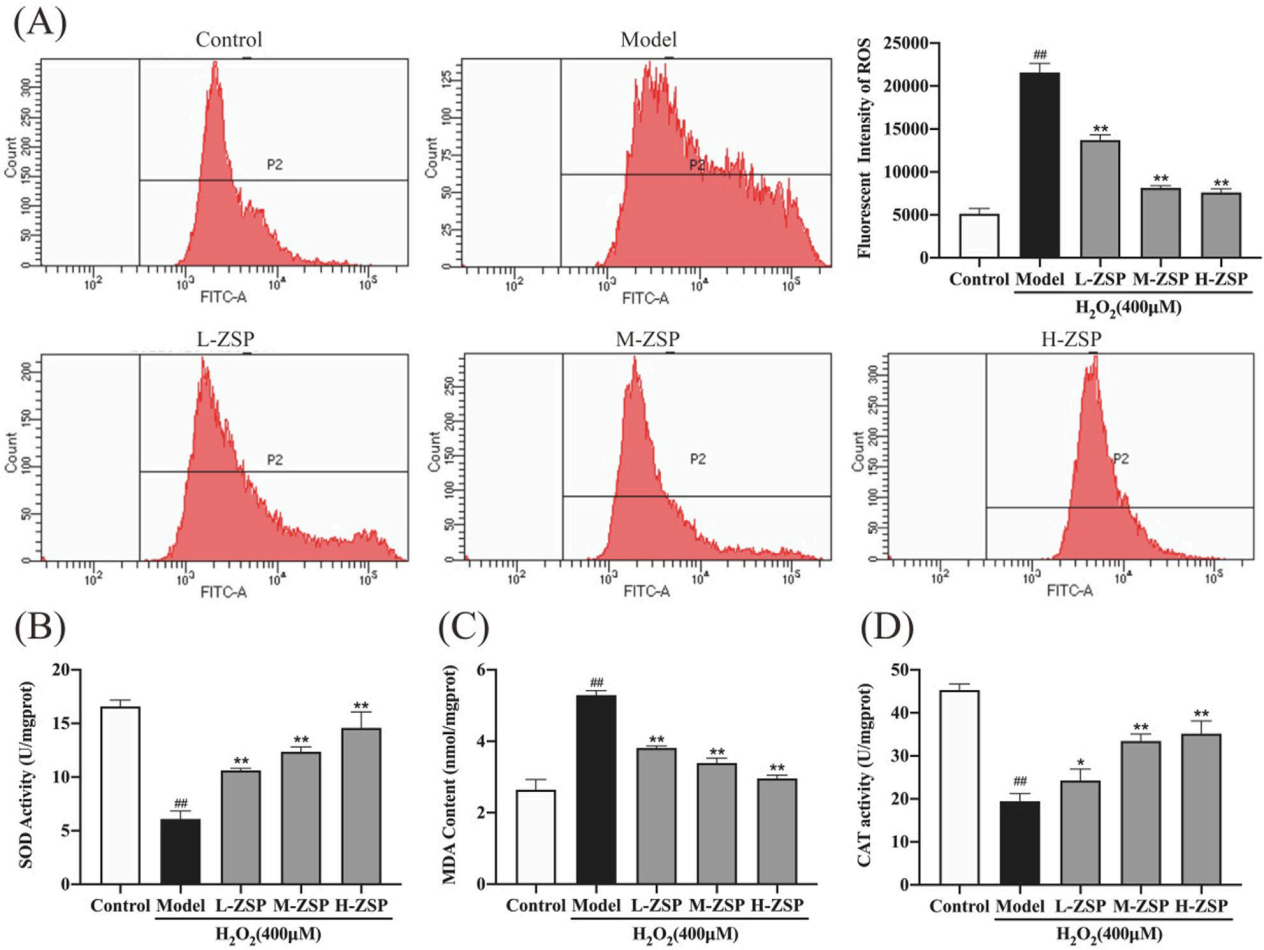
The influence of ZSP-containing serum on H_2_O_2_-induced oxidative stress and intercellular ROS production in PC12 cells. **(A)** The mean fluorescence intensities of ROS in each group. **(B)** SOD activity of PC12 cells in each group. **(C)** MDA content of PC12 cells in each group. **(D)** CAT activity of PC12 cells in each group. n = 3 per group. #*p* < 0.05 and ##*p* < 0.01, *versus* the control group. *p < 0.05 and **p < 0.01, *versus* the model group.

Exposure to 400 μM H_2_O_2_ significantly elevated the ROS levels in PC12 cells (Figure 2A). However, pretreatment with ZSP-containing serum effectively reduced intracellular ROS accumulation, with the most pronounced reductions observed in the medium-dose and high-dose groups. Moreover, H₂O₂ exposure resulted in a significant reduction in SOD and CAT activities, accompanied by an increase in MDA content in PC12 cells. These adverse effects were partially mitigated by ZSP-containing serum, with the most pronounced reversal observed at higher doses (Figures 2B–D). These findings fully suggest that ZSP-containing serum significantly restores the activity of intracellular antioxidant enzymes, inhibits lipid peroxidation, and attenuates oxidative damage. This indicates its potential therapeutic efficacy in modulating oxidative stress and providing cellular protection.

### 3.3 ZSP-containing serum prevented H_2_O_2_-induced mitochondria damage and apoptosis in PC12 cells

**FIGURE 3:**
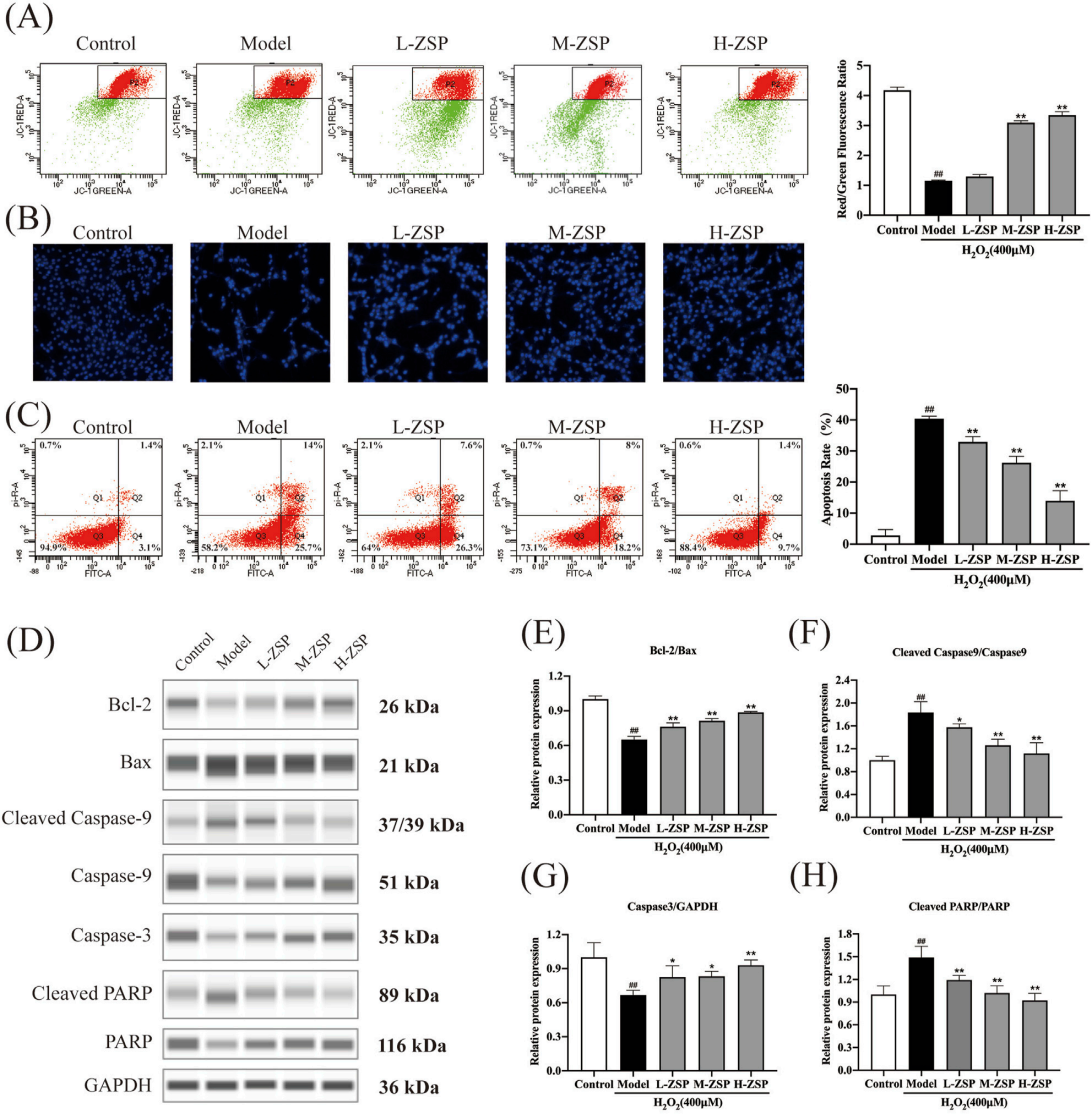
ZSP-containing serum prevented H_2_O_2_-induced mitochondria damage and apoptosis. **(A)** The red-green fluorescence ratio analysis of mitochondrial depolarization. **(B)** Apoptosis detected by staining with Hoechst 33258. **(C)** The effects of ZSP-containing serum on the apoptosis induced by H_2_O_2_-induced were determined by Annexin V/PI double staining using flow cytometry. **(D–H)** Representative Western blot bands and relative protein expression of Bcl-2, Bax, Caspase-3, cleaved Caspase-9, cleaved PARP and GAPDH. n = 3 per group. ^#^ *p* < 0.05 and ^##^ *p* < 0.01, *versus* the control group. ^*^ *p* < 0.05 and ^**^ *p* < 0.01, *versus* the model group.

The MMP of PC12 cells significantly decreased following H_2_O_2_ exposure, indicating activation of the intrinsic apoptosis pathway. Treatment with ZSP-containing serum attenuated the decrease in MMP in a dose-dependent manner (Figure 3A). Hoechst staining revealed uniform blue fluorescence in control PC12 cells, whereas H_2_O_2_-treated cells exhibited hyperchromatic and densely fluorescent particles within the apoptotic nuclear cytoplasm, indicative of pronounced cell apoptosis. Pretreatment with ZSP-containing serum reduced nuclear condensation and densely stained granular fluorescence in PC12 cells (Figure 3B). Consistent with these findings, flow cytometry analysis showed that ZSP-containing serum prevented neuronal apoptosis and increased cell survival rates (Figure 3C).

To further elucidate the anti-apoptotic effects of ZSP-containing serum, the expression of apoptosis-related proteins was examined (Figures 3D–H). Western blot analysis revealed that H_2_O_2_ exposure significantly decreased the Bcl-2/Bax ratio, an effect that was substantially reversed by ZSP-containing serum (Figures 3D,E). Additionally, ZSP-containing serum markedly inhibited the upregulation of caspase-3, caspase-9, and PARP expression in H_2_O_2_-induced PC12 cell injury (Figures 3F–H). These results demonstrate that pretreatment with ZSP-containing serum reduces apoptosis in a dose-dependent manner, mitigates the decline in mitochondrial membrane potential, and inhibits apoptosis by modulating the expression of Bcl-2, Bax, caspase-9, caspase-3, and PARP-related apoptosis proteins.

### 3.4 ZSP-containing serum pretreatment regulated the MAPK pathway in H_2_O_2_-treated PC12 cells

**FIGURE 4:**
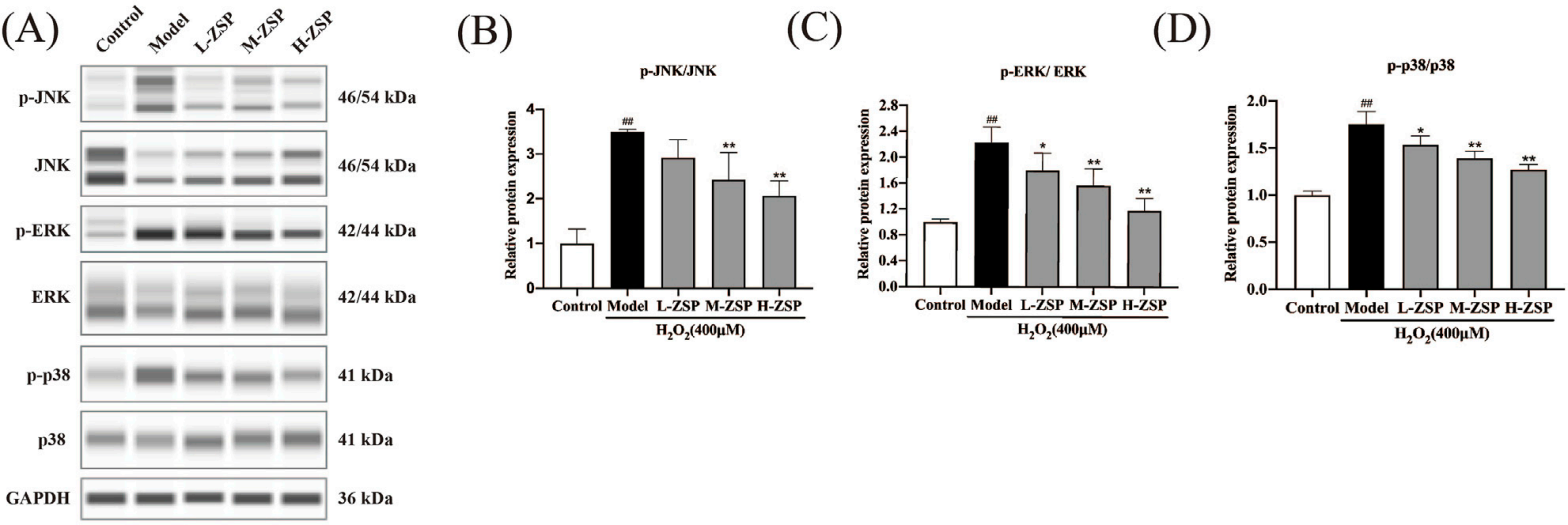
ZSP-containing serum pretreatment regulated MAPK pathway in PC12 cells after H_2_O_2_ injury. **(A–D)** Representative Western blot bands and relative protein expression of p-JNK, JNK, p-ERK, ERK, p-p38, p38 and GAPDH. n = 3 per group. #*p* < 0.05 and ##*p* < 0.01, *versus* the control group. *p < 0.05 and **p < 0.01, *versus* the model group.

Activation of JNK, ERK, and p38 was notably increased in PC12 cells of the model group. However, preincubation with ZSP-containing serum for 12 h significantly inhibited H_2_O_2_-induced activation of JNK, ERK, and p38, with the more pronounced effects observed in the medium-dose group and the high-dose group (Figure 4). These results suggest that the antioxidant and anti-apoptotic effects of ZSP-containing serum pretreatment on H₂O₂-damaged PC12 cells may be attributable to the inhibition of phosphorylation of key proteins within the MAPK signaling pathway, including JNK, ERK, and p38.

## 4 Discussion

PC12 cells, when highly differentiated, exhibit multiple processes of varying lengths and long synapse-like structures akin to neuronal axons, closely resembling the phenotype of nerve cells. These cells also possess the advantage of rapid proliferation, making them widely utilized in the study of neurodegenerative diseases such as stroke, Alzheimer’s disease, and Parkinson’s disease (Huang et al., 2023; Long et al., 2023; Wiatrak B et al., 2020; Sun Y et al., 2017). Consequently, highly differentiated PC12 cells were chosen for this research. The primary inducers for establishing cellular oxidative damage models include H_2_O_2_, t-BHP, and glutamate, among these various modeling methods, H_2_O_2_ is the most widely employed due to its accessibility and relatively stable properties. Exogenous H_2_O_2_ readily penetrates the cell membrane and enters the cell, where it forms highly reactive free radicals that induce cellular damage or apoptosis (Li WG et al., 2001). Consequently, this study employed H_2_O_2_ as an inducer to establish a PC12 cell oxidative damage model. Our experimental results indicated that at an H_2_O_2_ concentration of 400 μM, the cell survival rate was 60.94%, indicating moderate damage to PC12 cells. Thus, this concentration was deemed optimal for modeling purposes. This choice aligns with the H_2_O_2_ modeling concentration used in other experiment with PC12 cells (Lin P et al., 2015). By administering varying doses of ZSP, it is possible to determine its dose-dependent effects on cells. This approach aids in understanding both the efficacy and potential toxicity of the drug. Consequently, our experiments were designed to include low, medium, and high doses for pre-treatment of the cells. We determined the optimal concentration of H₂O₂ for inducing PC12 cell damage and successfully established an *in vitro* cell model of oxidative stress post-simulated IS. Concurrently, we prepared various doses of ZSP-containing serum, ultimately determining that a 5% concentration was optimal for subsequent experimentation.

To further investigate the protective and antioxidant effects of ZSP-containing serum on H_2_O_2_-induced damage in PC12 cells, we initially examined the cell morphology using microscopy. In the model group, the number, morphology, adhesion ability, and refractive index of PC12 cells were significantly impaired compared to the control group. Notably, the pre-treatment groups with ZSP-containing serum exhibited substantial improvement in cell condition. Particularly, the cell morphology in the high-dose group closely resembled that of the normal control group. LDH is a lactate dehydrogenase, it cannot penetrate the cell membrane. When cells are injured or dead, the integrity of cell membrane is impaired, leading to the release of LDH outside the cell (Adan et al., 2016). The existing results demonstrated that the oxidative damage to PC12 cells caused the release of LDH, and the ZSP treatment could reduce the release of LDH to reverse the trend, indicating a pronounced protective effect.

Oxidative stress stimulates superabundant accumulation of ROS in PC12 cells, and the pretreatment with ZSP-containing serum can alleviate the cumulation. MDA is an product of lipid peroxidation, and when stimulated by excessively oxidative reaction, the intracellular content of MDA will increase obviously (Valko et al., 2007). Thus, MDA is considered as an indicator of oxidative stress. Furthermore, there are various ROS scavenging enzymes in the organism, the most important of which are SOD and CAT (Chance et al., 1979). According to the consequences in our experiments, the MDA content increased after H_2_O_2_ exposure, accompanied by the decrease of SOD and CAT activities. The ZSP-containing serum pretreatment could lowered the MDA level, and increase the activities of SOD and CAT in the H_2_O_2_-induced PC12 cell injury dose-dependently.

Although neuroprotective interventions are urgently required during IS, the precise mechanisms of neuronal death remain unclear. Multiple pathways, including endogenous and exogenous apoptosis, necrosis, autophagy, ferroptosis, phagocytosis, and pyroptosis, may be involved (Bursch et al., 2000). Apoptosis is the most common form of programmed cell death in multicellular organisms. In the intrinsic pathway of apoptosis, the release of pro-apoptotic substances from the mitochondrial membrane space is crucial and is triggered by changes in MMP. Our study demonstrated that pretreatment with ZSP-containing serum improved nuclear morphology and reduced the apoptosis rate in H_2_O_2_-induced PC12 cell damage. The MMP of PC12 cells exposed to H_2_O_2_ was significantly reduced. However, pretreatment with ZSP-containing serum effectively prevented this reduction and protected PC12 cells from H_2_O_2_-induced oxidative stress.

The Bcl-2 protein family members play important roles in controlling apoptotic cell death and regulating mitochondrial permeability through mutual polymerization and depolymerization (Kaloni et al., 2023). The activities of anti-apoptotic and pro-apoptotic proteins are related to mitochondrial function and ROS concentration. Mitochondria trigger various signaling pathways by producing excessive ROS, including PI3-K, MAPK, p53 signaling pathways, etc (Niizuma et al., 2010). The caspase family consists of aspartate-specific cysteine proteolytic enzymes that exist in the cytoplasm. Their active sites contain cysteine residues, which can specifically cleave the peptide bond following the target aspartate residues, thereby activating or deactivating the target protein (Yamashima, 2000). H_2_O_2_ treatment could activate the intrinsic apoptosis pathway because the expression of Bcl-2/Bax was significantly downregulated, accompanied by the activation of Caspase-9 apoptotic proteins and PARP proteins and the deactivation of Caspase-3. However, the expression of Bcl-2/Bax was upregulated, the activation of Caspase-9 and PARP was inhibited, and the level of Caspase-3 was upregulated after pretreating PC12 cells with ZSP-containing serum.

ROS-induced oxidative stress can activate the MAPK signal pathway through changes in phosphorylation levels of JNK, ERK and p-38 (Fakhri et al., 2022). Various traditional prescriptions can achieve protective effects against cellular oxidative damage and apoptosis by regulating MAPK pathway (Jia et al., 2021; Zhu et al., 2019; He et al., 2019). Our data showed that the MAPK pathway in PC12 cells was overactivated by H_2_O_2_ exposure, and the ratios of p-JNK/JNK, p-ERK/ERK and p-p38/p38 expression levels were upregulated. However, pretreatment with ZSP-containing serum significantly reversed the activation of the MAPK pathway induced by H_2_O_2_ treatment. These results indicate that ZSP has neuroprotective effect against H_2_O_2_-induced oxidative damage, and the underlying mechanism of its anti-oxidation and anti-apoptotic actions may be correlated with the regulation of MAPK pathway.

While our study provides strong evidence, several limitations must be acknowledged. First, our experiments were conducted using *in vitro* cell models, and the results may not fully represent the *in vivo* situation. Second, we did not investigate the specific active ingredients of ZSP and their respective mechanisms of action, necessitating further research for clarification. Additionally, studies using animal models of IS have demonstrated that a moderate dose of ZSP is more effective than both high and low doses (Song Q et al., 2023), which differs from our findings. This discrepancy may be attributed to the complex physiological environment in animal models, where drug effects are influenced by numerous factors such as the immune system and hemodynamics, whereas the cell model is relatively simple and less affected by environmental factors. In animal models, high-dose drugs may undergo faster metabolism, resulting in effective concentrations similar to those of moderate doses. At high doses, metabolic enzymes may be induced, leading to increased drug clearance and thereby equating the effective concentrations to those of moderate doses. This involves drug metabolism and requires further investigation. Alternatively, the saturation of drug effects may be a factor. In animal models, moderate doses may achieve maximal effect, rendering higher doses ineffective in further enhancing the therapeutic outcome. Once the drug reaches a certain concentration, receptor occupancy may be complete, leading to saturation of drug efficacy.

This study is the first to elucidate the protective effects of ZSP-containing serum against H_2_O_2_-induced injury in PC12 cells and its potential mechanisms. Future research should aim to identify and characterize the specific active components of ZSP and evaluate their effects in in vivo models. Additionally, investigating the synergistic effects of ZSP with other neuroprotective agents will be an intriguing and valuable direction for further study.

## 5 Conclusion

Mongolian medicine ZSP provides protection to PC12 cells against H_2_O_2_-induced oxidative stress and apoptotic injury. The underlying mechanisms may involve inhibition of MAPK signaling pathway activation, enhancement of antioxidant enzyme activity, reduction of intracellular lipid peroxidation, and suppression of intrinsic apoptosis.

## Data Availability

The original contributions presented in the study are included in the article/Supplementary Material, further inquiries can be directed to the corresponding author.

## Glossary

ATCC: American Type Culture Collection
Bax: BCL2-Associated X
Bcl-2: B-cell lymphoma-2
Caspase: Cysteinyl aspartate specific proteinase
CAT: Catalase
DMEM: Dulbecco’s modified eagle medium
DMSO: Dimethyl sulfoxide
EDTA: EthyleneDiamine Tetraacetic Acid
ERK: Extracellular signal-regulated kinase
FBS: Fetal Bovine Serum
I/R: Ischemia/Reperfusion
IS: Ischemic stroke
JNK: c-Jun N-terminal kinase
LDH: Lactate dehydrogenase
MAPK: mitogen-activated protein kinase
MDA: Malondialdehyde
MMP: Mitochondrial membrane potential
OD: Optical Density
p38 MAPK: p38 mitogen-activated protein kinase
PARP: Poly ADP-ribose polymerase
PBS: Phosphate buffer saline
PC12: Pheochromocytoma Cells
PI: Propidium lodide
ROS: Reactive oxygen species
SOD: Superoxide dismutase
ZSP: Zhachong Shisanwei Pill
